# Nutritional profile of Indian vegetarian diets – the Indian Migration Study (IMS)

**DOI:** 10.1186/1475-2891-13-55

**Published:** 2014-06-04

**Authors:** Krithiga Shridhar, Preet Kaur Dhillon, Liza Bowen, Sanjay Kinra, Ankalmadugu Venkatsubbareddy Bharathi, Dorairaj Prabhakaran, Kolli Srinath Reddy, Shah Ebrahim

**Affiliations:** 1 South Asia Network of Chronic Disease, Public Health Foundation of India, Building 47, Sector 44 Gurgaon, New Delhi, Haryana 122002, India; 2 London School of Hygiene and Tropical Medicine, London, UK; 3 Chief Nutritionist Just Right Obesity Clinic, Bangalore, India; 4 Centre for Chronic Disease Control, New Delhi, India; 5 Public Health Foundation of India, New Delhi, India

**Keywords:** India, Diet, Nutrition, Vegetarian, Vitamin B12

## Abstract

**Background:**

The cardiovascular and other health benefits and potential harms of protein and micronutrient deficiency of vegetarian diets continue to be debated.

**Methods:**

Study participants included urban migrants, their rural siblings and urban residents (n = 6555, mean age - 40.9 yrs) of the Indian Migration Study from Lucknow, Nagpur, Hyderabad and Bangalore. Information on diet (validated interviewer-administered semi-quantitative food frequency questionnaire), tobacco, alcohol, physical activity, medical histories, as well as blood pressure, fasting blood and anthropometric measurements were collected. Nutrient databases were used to calculate nutrient content of regional recipes. Vegetarians ate no eggs, fish, poultry and meat. Using multivariate linear regression with robust standard error model, we compared the macro- and micro-nutrient profile of vegetarian and non-vegetarian diets.

**Results:**

Vegetarians, (32.8% of the population), consumed greater amounts of legumes, vegetables, roots and tubers, dairy and sugar, while non-vegetarians had a greater intake of cereals, fruits, spices, salt (p < 0.01), fats and oils. Vegetarians had a higher socioeconomic status, and were less likely to smoke, drink alcohol (p < 0.0001) and engage in less physical activity (p = 0.04). On multivariate analysis, vegetarians consumed more carbohydrates (β = 7.0 g/day (95% CI: 9.9 to 4.0), p < 0.0001), vitamin C (β = 8.7 mg/day (95% CI: 4.3 to13.0), p < 0.0001) and folate (β = 8.0 mcg/day (95% CI: 3.3 to 12.7), p = 0.001) and lower levels of fat (β = −1.6 g/day (95% CI: −0.62 to −2.7), p = 0.002), protein (β = −6.4 g/day (95% CI: −5.8 to −7.0), p < 0.0001), vitamin B12 (β = −1.4 mcg/day (95% CI: −1.2 to −1.5), p < 0.0001) and zinc (β = −0.6 mg/day (95% CI: −0.4 to −0.7), p < 0.0001).

**Conclusion:**

Overall, Indian vegetarian diets were found to be adequate to sustain nutritional demands according to recommended dietary allowances with less fat. Lower vitamin B12 bio-availability remains a concern and requires exploration of acceptable dietary sources for vegetarians.

## Introduction

In response to the growing burden of non-communicable diseases (NCDs), the World Health Organization (WHO) recommends a reduced intake of fat, sugar and salt, and a higher intake of fruits, vegetables, whole grains and nuts, while maintaining energy balance and healthy weight [1]. The vegetarian diet [2,3], among others such as the Dietary Approaches to Stop Hypertension (DASH), Mediterranean and Japanese diets, may offer benefits for reducing risk of NCDs [2,4]. Evidence on the health benefits of a vegetarian diet from long-term cohort studies in the West, such as EPIC-OXFORD and the Adventist Health study II, show positive cardiovascular, cancer, mental health and overall mortality effects [5-9]. However, vegetarian diets may result in inadequate nutritional intake of omega-3 fatty acids, vitamin B_12_, protein and minerals, such as iron and zinc [10-12] due to reduced bio-availability in plant sources. Reduced vitamin B12 and omega-3 fatty acids are also associated with increased serum levels of homocysteine and aggregation of platelets that contribute to cardiovascular disease [13,14].

In Western populations, vegetarianism is usually an adopted life-style by choice during adulthood [15]. Moreover, vegetarianism is not common in the West (<5%) [16], which limits the power of studies examining the macro- and micro-nutrient value of vegetarian diets [15-18]. By contrast, a substantial proportion of the Indian population (35%) are vegetarians [16] (10- 62% for different regions) [19]. Dietary patterns in India are bound by religious, cultural and family values [20], are often maintained for generations, and not necessarily associated with other healthy lifestyle behaviors, such as increased physical activity. These differences provide opportunities to make more robust evaluations of the nutritional profile of vegetarian diets in India. The vegetarian diet in India includes a wide range of vegetables, fruits, cereals, pulses, spices, seasonings and cooking practices [20] and hence can have different levels of bio-availability and absorption for many nutrients. Thus, information on the nutritional profile of vegetarian diet across various regions of India is a valuable addition to the existing body of evidence.

Hence in this paper we present a comparative analysis of macro- and micro-nutrient profiles of vegetarian and non-vegetarian diets from four geographic regions of the country.

## Methods

### Subjects and study design

The Indian Migration Study (IMS) is a sib-pair study nested within the larger Cardio-Vascular Disease Risk Factor Study (CVDRFS) in industrial populations from 10 companies across India (n = 19, 973 for the questionnaire survey, n = 10, 442 for biochemical investigations) [21]. Details of the study design and methods have been reported earlier [22,23]. In brief, the IMS was carried out in four city factory settings (out of 10 in CVDRFS) from north (Lucknow, Hindustan Aeronautics Ltd), central (Nagpur, Indorma Synthetics Ltd) and southern India (Hyderabad, BHEL; and Hindustan Machine tools Ltd) from 2005 to 2007. Factory workers, their rural siblings and co-resident spouses who had migrated from rural to urban areas, along with a 25% random sample of urban non-migrants and their co-resident spouses, were asked to participate in the study. Of the 7,594 migrant and non-migrant factory workers and their co-resident spouses eligible for the Indian Migration Study, 7,102 (94%) agreed to complete the clinical examination with their sibling, of whom 3,537 sib- pairs participated and the final IMS sample included 7,067 respondents.

### Data collection

An interviewer–administered questionnaire was used to collect socio-demographic, diet, physical activity and health data from the respondents. A Standard of Living Index (SLI) was derived using a sub-set of questions on socio-economic position such as quality of house, toilet facilities, land ownership, sources of lighting and drinking water and possession of household articles (14 items). Physical activity was assessed in the past month for leisure time, occupation and other common daily activities [24]. Frequency and average duration in minutes of each activity undertaken were collected. Metabolic Equivalent tasks (METs) were estimated as the rate of resting metabolic rate where 1 MET is equivalent to the energy expenditure value of sitting quietly using an established method [24].

### Dietary assessment

Diet was assessed using a validated interviewer-administered semi-quantitative food frequency questionnaire (FFQ) [25]. The FFQ collected information on portion size and frequency of 184 commonly consumed food items over the last one year. Standard portion size (e.g., tablespoon, ladle, and bowl) and frequency (daily, weekly, monthly, yearly/never) were recorded after showing examples of vessels and different portion size. A single FFQ was designed to cover the four main regions of the study. A nutrient database was developed for the FFQ by obtaining recipes for each item from participants who volunteered to prepare the recipes under a nutritionist’s supervision; separate recipes were collected for each food item based on location (four sites; Lucknow, Nagpur, Hyderabad and Bangalore) and regional migration status (urban/rural/migrant). Nutrient databases were used to calculate the macro and micro-nutrient content of each recipe using Indian food composition tables [26] and the United States Department of Agriculture nutrient database (USDA, Release No. 14) [27] or McCance and Widdowson’s Composition of Foods [28], where nutrient values were unavailable from the Indian food composition tables. Energy, protein, fat, fibre for macro-nutrients and iron, calcium, zinc, folate, Vitamin C and B12 for micro-nutrients were calculated. Recipes were also used to generate databases of the food group composition of each food item, and used to calculate average daily food group intake. Three 24-hour recalls were implemented in a sub-sample of participants (n = 530, 53.9% male) to validate the FFQ. A sub-sample was re-interviewed after completion of the FFQ (1–2 months, n = 185 and 12 months later, n = 305), yielding kappa coefficients = 0.26-0.71 [25], which are similar to reliability estimates from other studies [29,30]. The energy adjusted spearman correlation coefficients for macro-nutrients ranged from 0.43 (fibre) to 0.52 (fats) based on comparisons of FFQ with 24-hour recalls [25]. Vegetarians were classified as those who ate no eggs, meat, fish and poultry which is the most common form of vegetarianism in India (lacto-vegetarians) [31]; persons who ate eggs in their diet (lacto-ovo-vegetarians (n = 215); <5%) were excluded due to small numbers. The estimated macro- and micro-nutrient levels among vegetarians and non-vegetarians were compared to the Recommended Dietary Allowance (RDA) according to Indian standards.

### Physical and biochemical parameters

A digital personal scale (Beurer Model PS16, Ulm, Germany) and stadiometer accurate to 1 mm (Leicester height measure; Chasmors Ltd London UK) were used by trained personnel to record the weight and height respectively of the participants in light indoor clothes without shoes. Blood pressure was measured on the right upper arm with the participant in the sitting position after a rest of 5 minutes. Two readings were taken using an appropriate-sized cuff connected to a digital device (model M5-I; Omron-Matsusaka Company, Matsusaka City, Japan). Skin fold thickness was measured three times at triceps, subscapular and median calf using Holtain calipers and the average of three measurements. Triceps and subscapular measurements were used to calculate the percent body fat using standard formula [32]. Fasting (>8 hours) blood samples were collected and centrifuged immediately, stored locally at −20°C, and transported monthly to the All India Institute of Medical Sciences (AIIMS), New Delhi for biochemical assays and for storage at −70°C.

### Statistical analysis

We excluded participants who had incomplete diet data (n = 1), who were ovo-vegetarians (n = 215), who had incomplete physical activity data (n = 70) and/or whose estimated energy levels were unacceptable (<500 kcal (n = 2) and >5000 kcal (n = 224)). The final analysis was carried out in 6,555 participants.

Socio-demographic characteristics, life-style risk factors, and cardio-vascular outcomes were compared among vegetarians and non-vegetarians using the t-test and chi-square test for continuous and categorical data, respectively. To account for the skewed nature of food consumption patterns, non-parametric tests (Wilcoxon Rank sum test) were used for comparing the distribution of micro- and macro- nutrient levels in vegetarians and non-vegetarians.

To account for the correlated nature of sib-pair comparisons, multivariate linear regression model with robust standard errors were used [33], which accounted for the sib-pair clusters in evaluating the association of vegetarian and non-vegetarian diets and nutrient levels. Multivariate analyses included the adjustment of important confounders such as age (continuous, years), gender (male/female), socioeconomic status (standard of living index, 1–36), energy (quartiles, in kcal) and physical activity (quartiles, total METs). All statistical analyses were conducted using STATA software version 10 (StataCorp.2009.Stata Statistical Software: Release 10. StataCorp LP).

### Ethics approval

Information sheets in local language were given to the participants and their signatures were obtained in the consent forms. Ethics committee approval was obtained from All India Institute of Medical Sciences Ethics Committee, reference number A-60/4/8/2004, and the procedures followed were in accordance with the ethical standards of the committee.

## Results

Of 6,555 eligible participants, 2,148 (32.8%) were vegetarians and 4407 (67.2%) were non-vegetarians. Among vegetarians, 56.5% were men and 43.3% were women and 33.2% lived in a rural region, 30.8% were migrants and 35.9% were urban dwellers. The prevalence of vegetarianism varied from 10.1% in Hyderabad to 47.5% at Luck now with Nagpur (23.8%) and Bangalore (18.4%) recording average values (data not shown). Vegetarians had a significantly higher standard of living (mean SLI (SD) = 23.0 (6.3) vs. 21.0 (6.7), p < 0.0001), were less likely to smoke (7.5% vs. 11.8%, p < 0.0001) or drink alcohol (5.7% vs. 21.3%, p < 0.0001) and more likely to be urban dwelling (35.9% vs. 31.3%, p < 0.0001). Non-vegetarians had greater physical activity levels (p = 0.04) and higher hemoglobin levels (p < 0.0001). There was no difference between vegetarians and non-vegetarians with respect to age, use of smokeless tobacco, body mass index (BMI), percent body fat, prevalence of diabetes and hypertension (Table 1).

**Table 1:** Socio-demographic and lifestyle characteristics of Indian migration study population

	**Vegetarians**	**Non-vegetarians**	p-value*
	**(n = 2148)**	**(n = 4407)**	
	**%/mean (SD)**	**%/mean (SD)**	
**Age (yrs)**	41.2(10.2)	40.8(10.4)	0.2
**Standard of living index +**	23.0(6.3)	21.0(6.7)	<0.0001
**Gender**			
Male	56.5	59.0	0.05
Female	43.5	41.0	
**Migrant status**			
Rural	33.2	38.2	<0.0001
Migrants	30.8	30.4	
Urban	35.9	31.3	
**Location**			
Luck now	47.5	19.6	<0.0001
Nagpur	23.8	21.4	
Hyderabad	10.1	37.6	
Bangalore	18.4	21.3	
**Smoking**			
Never	90.6	85.7	<0.0001
Previous	1.9	2.5	
Current	7.5	11.8	
**Tobacco chewing**			
Never	84.8	83.7	0.13
Previous	2.4	2.0	
Current	12.8	14.3	
**Alcohol**			
Never	91.9	75.1	<0.0001
Previous	2.2	3.4	
Current	5.7	21.3	
**Diabetes**			
No	90.4	89.8	0.48
Yes	9.7	10.2	
**Hypertension**			
No	76.1	74.6	0.20
Yes	23.8	25.3	
**Hemoglobin g/dl**	12.7(1.8)	13.3(3.7)	<0.0001
**Physical activity (METS ‡)**	38.6(4.2)	38.9(4.7)	0.04
**BMI kg/m**^2^	23.9(4.4)	23.9(4.5)	0.99
**%Body fat**	27.1(8.2)	27(8.2)	0.51

*p-values are from t-test of significance for continuous data and chi-square test of significance for categorical data.

+Standard of Living Index (SLI) distribution is 1–36 (Median 23, IQR =17-27).

**‡**Metabolic Equivalent tasks.

Vegetarians consumed greater amounts (p < 0.01) of legumes, vegetables, root and tubers, dairy and sugar while non-vegetarians had greater intakes of cereals, fruits, spices and salt (p < 0.01) as well as more fats and oils (Table 2). On multivariate analysis, regarding macro-nutrients, vegetarians consumed less total fat (β = −1.6 g/day (95% CI: −0.62 to −2.7), p = 0.002) and protein (β = −6.4 g/day (95% CI:-5.8 to −7.0), p < 0.0001) and more carbohydrate (β = 7.0 g/day (95% CI: 9.9 to 4.0), p < 0.0001) (Table 3). A multivariate sub-analysis based on different geographical locations and regions showed variations in fat and carbohydrate consumption, although vegetarians across all locations such as Lucknow, Nagpur, Hyderabad and Bangalore in urban, migrant and rural areas (p < 0.0001) consumed less protein (data not shown).

**Table 2:** Food consumption patterns of Indian migration study population

**Food group**	**Vegetarians**	**Non-vegetarians**
(g/day)	(n = 2148) (Median; IQR)	(n = 4407) (Median; IQR)
**Cereals**	366.9	372.1*
	(285.2-469.6)	(289.0-479.6)
**Legumes**	61.0	45.8**
	(42.0-85.2)	(29.3-69.7)
**Fruits**	115.4	126.0**
	(68.4-199.5)	(71.5-210.5)
**Vegetables**	241.8	199.6**
	(168.0-336.7)	(125.9-300.6)
**Roots tubers**	47.3	28.7**
	(23.4-86.4)	(11.3-57.4)
**Spices**	17.1	18.6**
	(12.5-22.5)	(13.2-25.4)
**Fish**	-	3.9
		(0.5-9.9)
**Meat & poultry**	-	20.3
		(9.6-38.9)
**Dairy**	352.8	291.2**
	(215.8-502.0)	(183.6- 431.7)
**Nuts**	10.1	10.0
	(6.1-17.3)	(5.5-18.6)
**Eggs**	-	0.1
**(number/day)**		(0.06-0.3)
**Fats & oils**	43.4	44.2
	(32.2-57.5)	(31.6-60.9)
**Sugar**	37.0	31.2**
	(25.8-50.9)	(20.6-45.2)
**Salt**	7.8	9.1**
	(5.8-10.9)	(6.6-12.3)

**p < 0.01 *p < 0.05 (Wilcoxon Rank Sum non-parametric test).

**Table 3:** Multivariate linear regression models* for macro- and micro-nutrient intake of Indian migration study population comparing non-vegetarian with vegetarian diets

**Macro–micro nutrient**	** Model I**		** Model II**		** Model III†**	
	**β (95% CI)**	**p-value**	**β (95% CI)**	**p-value**	**β (95% CI)**	**p-value**
**Protein** (g/day)	2.1 (0.8 to 3.5)	0.002	4.0 (2.7 to 5.3)	< 0.0001	6.4 (5.8 to 7.0)	<0.0001
**Total fat** (g/day)	1.8 (0.1 to 3.5)	0.03	3.9 (2.2 to 5.5)	< 0.0001	1.6 (0.62 to 2.7)	0.002
**Sat fat** (g/day)	−0.37 (−0.9 to 0.23)	0.22	0 .34 (−0.24 to 0.93)	0.25	−0.13 (−0.57 to 0.30)	0.54
**MUFA** (g/day)	−1.2 (−1.9 to −0.5)	<0.0001	−0. 73 (−1.4 to 0.05)	0.03	0.94 (0.40 to 1.4)	0.001
**PUFA** (g/day)	3.4 (2.6 to 4.1)	< 0.0001	4.1 (3.4 to 4.9)	< 0.0001	0.57 (0.02 to 1.1)	0.04
**Total fibre** (g/day)	−0.78 (−1.0 to −0.4)	< 0.0001	−0.41 (−0.70 to 0.11)	0.006	−0.15 (−0.34 to 0.03)	0.11
**Carbohydrate** (g/day)	−3.6 (−10.9 to 3.5)	0.3	1.0 ( −5.4 to 8.5)	0.66	−7.0 (−9.9 to −4.0)	<0.0001
**Iron** (mg/day)	−2.6 (−3.1 to −2.0)	< 0.0001	−1.8 (−2.4 to −1.3)	< 0.0001	−0.07 (−0.34 to 0.21)	0.64
**VitaminB12** (mcg/day)	2.0 (1.8 to 2.2)	< 0.0001	2.1 (1.9 to 2.3)	< 0.0001	1.4 (1.2 to 1.5)	<0.0001
**Calcium** (mg/day)	−18.3 (−43.0 to 6.4 )	0.14	16.4 (−7.4 to 40.3)	0.176	11.3 (−18.5 to 16.9)	0.92
**Zinc** (mg/day)	0.18 (−0.03 to 0.41)	0.10	0.49 (0.28 to 0 .71)	< 0.0001	0.55 (0 .44 to 0.65)	<0.0001
**Vitamin C** (mg/day)	−4.1 (−9.0 to 0. 68)	0.09	2.7 (−2.0 to 7.5)	0.08	−8.7 (−13.0 to −4.3)	< 0.0001
**Folate** (mcg/day)	−27.1 (−34.4 to −19.8)	< 0.0001	−15.8 (−22.8 to −8.8)	< 0.0001	−8.0 (−12.7 to −3.3)	0.001

†When models were run using log-transformed outcome variables, p-value significance levels remained the same except total fibre which became significant at p =0.01. β = for 1-unit increase of each nutrient comparing vegetarians and non-vegetarians.

*Robust Standard Error.

Reference group - Vegetarians.

Model I – Unadjusted; Model II- Adjusted for age, sex, SLI and Sib-Pair.

Model II- Adjusted for age, sex, SLI, site, migration status, energy, physical activity and Sib-Pair.

On multivariate analysis, vegetarians had higher consumption of micro-nutrients such as vitamin C (β = 8.7 mg/day (95% CI: 4.3 to 13.0), p < 0.0001) and folate (β = 8.0 mcg/day (95% CI: 3.3 to 12.7), p = 0.001) and lower consumption of vitamin B12 (β = −1.4 mcg/day (95% CI: −1.2 to −1.5), p < 0.0001) and zinc (β = −0.6 mg/day (95% CI: −0.4 to −0.7), p < 0.0001) than non-vegetarians (Table 3). A sub-analysis (multivariate) by region showed variations in vitamin C, folate and zinc consumption, although vegetarians across all locations - Lucknow, Nagpur, Hyderabad and Bangalore - in urban, migrant and rural populations (p < 0.0001) consumed less vitamin B12 (data not shown).

When we evaluated the IMS study population with respect to the Recommended Dietary Allowance (RDA), we found a higher proportions of vegetarians consuming protein (80.2% vs. 77.5%, p < 0.01) and micro-nutrients such as vitamin C (99.2% vs.98.0%, p < 0.01), iron (76.8% vs. 63.6%, p < 0.01), calcium (87.5% vs. 83.2%, p < 0.01) and folate (93.4% vs. 87.0, p < 0.01) at RDA levels than non-vegetarians with less total energy intake (median 2712.2 vs. 2728.8 kcal/day, p > 0.05). However, a greater proportion of vegetarians were below vitamin B12 RDA levels (35.1% vs. 12.6%, p < 0.01). Both vegetarians and non-vegetarians consumed less fibre than the RDA (86.7% of vegetarians and 87.0% of non-vegetarians) (Table 4) and a sub-analysis to explore the reason for fibre deficiency in both vegetarians and non-vegetarians revealed a strong association (p < 0.0001) with socio-economic status (data not shown).

**Table 4:** Macro and micro-nutrient intake (estimated) of Indian migration study population in comparison to RDA

**Macro–micro nutrient**	**Vegetarians (V)**	**Non-vegetarians (NV)**	**% below RDA#**		**RDA**	
**(Median; IQR)**	**(n = 2148)**	**(n = 4407)**	**V**	**NV**	**Men**	**Women**
**Energy** kcal/day	2712.2 (2235.8-3321.7)	2728.8 (2156.5-3433.3)	38.7	40.5	2730 (for moderate work)	2230 (for moderate work)
**Protein** g/day	76.1 (60.8-92.8)	78.1* (59.9-97.7)	19.8	22.5*	60.0	55.0
**Carbohydrate** g/day	431.5 (351.8 to 526.4)	426.0 (340.3 to 533.5)	NA	NA	NA	NA
**Fibre** g/day	13.7 (10.7-17.3)	12.8** (9.3-17.0)	86.7	87.0	20.0	20.0
**Total fat** g/day	74.2 (58.9-95.7)	75.9 (57.5-101.1)	NA	NA	NA	NA
**Sat fat** g/day	22.9 (17.1-29.9)	22.0** (15.9-30.1)	NA	NA	NA	NA
**MUFA** g/day	23.8 (16.9-31.9)	22.1** (15.8-30.3)	NA	NA	NA	NA
**PUFA** g/day	18.6 (12.4-29.7)	23.1** (14.7-34.1)	NA	NA	NA	NA
**Iron** mg/day	25.5 (19.2-32.5)	22.4** (15.6-30.9)	23.2	36.4**	17.0	21.0
**Calcium** mg/day	980.6 (751.0-1247.1)	946.5** (692.9-1253.1)	12.5	16.8**	600.0	600.0
**Zinc** mg/day	11.6 (8.9-14.5)	11.6 (8.8-14.9)	43.2	45.4	12.0	10.0
**Vitamin C** mg/day	142.7 (100.7-200.5)	136.9** (92.3-197.4)	0.8	2.0**	40.0	40.0
**Vitamin B12** mcg/day	1.2 (0.8-1.8)	2.2** (1.3-3.6)	35.1	12.6**	1.0	1.0
**Folate** mcg/day	355.6 (279.4-436.3)	327.2** (247.0-419.4)	6.6	13**	200.0	200.0

**p < 0.01; *p < 0.05; NA - Not Applicable.

Wilcoxon Rank Sum non-parametric test for continuous data and Chi-square test for categorical data.

#% below RDA is a single variable which includes RDA for men and women separately wherever required.

A descriptive analysis of macro- and micro- nutrient consumption of vegetarians and non-vegetarians based on geographical location and migration status further showed that vegetarians consumed lower levels of protein on average than non-vegetarians, regardless of location and migration status. Except for rural and migrant populations in Lucknow, the median energy intake in vegetarians was lower than non-vegetarians. No clear pattern was observed in the consumption of carbohydrates, fat and fibre. Similarly vegetarians consumed less vitamin B12 across all regions and locations than non-vegetarians and there was no pattern evident in the consumption of other micro-nutrients except for zinc (Additional file 1: Table S1).

## Discussion

We found positive effects of vegetarian diet compared to the non-vegetarian diet in terms of food consumption patterns and nutrient intake across four geographic regions and diets of India. Vegetarians consumed greater amounts of legumes, vegetables, roots and tubers, dairy and sugar, while non-vegetarians had greater cereals, fruits, spices, salt, fats and oils. Vegetarians consumed greater amounts of carbohydrates, vitamin C and folate and less fat, protein, vitamin B12 and zinc than non-vegetarians. The lower fibre intake by both vegetarians and non-vegetarians may be confounded by socioeconomic status in our study population.

RDA comparisons indicated that a greater proportion of vegetarians were consuming adequate amounts of protein and micro-nutrients (iron, calcium, vitamin C and folate) and also consumed less total energy than non-vegetarians in different regions and locations. Our study is in contrast to findings of a lower intake of protein among vegetarians that has been found by other studies [12,34], although a recent study of Buddhist vegetarians found a compensatory increase in protein from plant sources resulting in a higher overall protein intake in vegetarians than in non-vegetarians [11]. Recent systematic review [35] and meta-analysis [36] have also indicated possible benefits of plant proteins for cardiovascular health.

We also found a sufficient intake of iron in vegetarians compared to non-vegetarians, which is similar to some [11,37,38] but not all previous studies [39,40]. One explanation is that non-heme iron is found in abundance in plant sources such as legumes, roots and tubers and their bio-availability is increased with concomitant intake of vitamin C-rich diet [10,38,39,41]. Other explanations for increased bio-availability of non-heme iron include baking chappathis in iron plates and the addition of ascorbic acid to cereals and pulses [42,43]. However a study on young women from Bangalore (India) estimates that only 2.8% of iron (non-heme) is available from plant sources [44].

Vegetarians develop vitamin B12 deficiencies compared to non-vegetarians due to the reduced bio-availability from plant sources [10,45]. Our findings are consistent with previous evidence showing a 4.4-fold increase in B12 deficiency in vegetarians among 441 rural and urban men in Pune (India) [46] and an international study on South Asians showing a 2.7 fold difference in deficiency (24% vs. 9% in non-vegetarians) [47]. Recent systematic review of homocysteine status (a marker of B12 levels) revealed significantly lower serum mean levels of vitamin B12 in vegetarians (lacto- or lacto-ovo) compared to non-vegetarians (209 ± 47 pmol/L vs. 303 ± 72 pmol/L, p < 0.005) [17]. The deficiency of vitamin B12 associated with vegetarian diet has been consistently established by studies conducted both in India [14,45,46,48] and outside India [17], and also suggest a role for gene polymorphisms associated with diet and defective absorption of vitamin B12 [14,49-51].

In our study, vegetarians consumed significantly less zinc compared to non-vegetarians (p < 0.0001), which is due to the lower bio-availability of zinc from plant sources [39]. Our findings that a greater proportion of vegetarians consumed both macro- and micro- nutrients at RDA levels compared to non-vegetarians (except for vitamin B12) and less total energy is similar to the other studies outside India [34,52-54]. In part, this may be due to higher socio-economic levels of vegetarians in our population which is similar to results from the National Family Health Survey-3 (NFHS-3, India), in which a greater proportion of vegetarians were in the highest wealth quintile compared to non-vegetarians (32.5% vs. 19.8%, p < 0.0001) [19].

The nutritional profile of vegetarian and non-vegetarian diet across various regions of India is not well documented [55], although studies have shown greater amounts of anti-oxidants (vitamin C, A, E) in Indian vegetarians that may make them less prone to oxidative stress and NCDs [56,57]. However, some studies on the nutritional profile of Indian vegetarian diet, demonstrate micro-nutrient deficiencies of zinc and iron that are primarily due to reduced absorption [58,40] and vitamin B12 deficiency in rural and urban vegetarians due to low dietary intake [46,48]. Our study differs from previous studies in India, as a large study population from 4 different geographical regions, representing 20 states of Indian, with energy- and multivariate adjusted analyses of nutritional intake. This could also be the reason for the differences observed in certain macro- and micro-nutrients consumption pattern between univariate (Table 4) and multivariate (Table 3) comparisons.

One limitation in our study is the possible over-estimation of certain nutrients intake by FFQ which could have led to a little over-estimation of percent population (vegetarian and non-vegetarian) meeting the RDA and the inability to capture the seasonal variation of fruit and vegetable intake, although the total time period (such as number of months in a year) of consumption of fruits and vegetables was captured. To assess the validity of the FFQ it was re-administered to 530 factory workers and rural dwellers, followed by three 24 hour recalls on different days. Nutrient and food group intake calculated by these two methods were compared using medians, kappa statistics, and Bland- Altman plots. The results demonstrated the validity and feasibility of measuring dietary intake in across various regions of India with a single FFQ [25].

Another limitation is that macro- and micro- nutrients estimation was done using food composition tables and does not account for the moisture content or cooking loss of the nutrients. However, even though vitamins undergo 25-40% loss during cooking [59], Indian food composition tables can be used to make adequate estimates of macro-nutrients and micro-nutrients for population/group level comparisons [33,60]. We also accounted for the sibling pair study design by using robust standard error model. The exclusion of unacceptable energy levels of the participants (<500 kcal or >5000 kcal) addressed the skewed nature of the data generally seen with the nutrient level estimation. Moreover, we ran multivariate models based on log-transformed outcome variables and the findings remained the same.

Another potential limitation is that vegetarians in our study population (industrial workers and their rural siblings) had a higher standard of living in both urban and rural populations than non-vegetarians, which may influence the results. However, our large, diverse sample represented four different geographical regions of India, in both urban and rural populations, and NFHS-3 data suggest that our comparison groups are representative with respect to socioeconomic status. More in-depth studies using more detailed repeated 24-hour dietary assessments on representative populations are required to corroborate our findings. Longitudinal studies are needed to explore the health consequences of long-term vegetarian compared to non-vegetarian diets.

## Conclusion

Overall, Indian vegetarian diets were found to have a greater percentage meeting RDA levels of macro- and micro- nutrients with less fat and lower calories than non-vegetarian diets. Vitamin B12 bio-availability remains a concern and should be addressed by exploring various dietary patterns associated with deficiency across various regions of India and identifying people who need supplementation. More elaborate studies assessing the cooking patterns in different regions and the nutritional loss during cooking are also required.

## Abbreviations

NCDs: Non-communicable diseases; WHO: World health organization; DASH: Dietary approaches to stop hypertension; IMS: The Indian migration study; CVDRFS: Cardio-vascular disease risk factor study; SLI: Standard of living index; METs: Metabolic equivalent tasks; FFQ: Food frequency questionnaire; RDA: Recommended dietary allowance; BMI: Body mass index; NFHS-3: National family health survey-3.

## Competing interests

The authors declare that they have no competing interests.

## Authors’ contributions

SE, DP, KSR, SK, AVB and LB contributed to the conception and design of the study. KS and PKD contributed to the analysis and interpretation of data and drafting the manuscript. PKD and SE contributed to critical revision of the paper. All authors have read the manuscript and gave their final approval for this version of the manuscript to be published.

## Supplementary Material

Additional file 1: Table S1 Macro and micro-nutrient intake (estimated) of Indian Migration Study population based on geographical location and migration status. Click here for file
