# Supplementary material for: Nutritional profile of Indian vegetarian diets – the Indian Migration Study (IMS)
Source: Nutr J. 2014 Jun 4;13:55. doi: 10.1186/1475-2891-13-55 (PMC4055802; doi:10.1186/1475-2891-13-55)
Supplement: Additional file 1: Table S1 — Macro and micro-nutrient intake (estimated) of Indian Migration Study population based on geographical location and migration status. [file 1475-2891-13-55-S1.docx]

| **Macro-Micro**  **Supplemental Table I** : Macro and micro-nutrient Intake (estimated) of Indian Migration Study population based on geographical location and migration status  **nutrient**  (Median; IQR) | **Lucknow**  **(n=1,886)**  **Median (IQR)** | | | | | | **Nagpur**  **(n= 1,457)**  **Median (IQR)** | | | | | | **Hyderabad**  **(n=1,876)**  **Median (IQR)** | | | | | | **Bangalore**  **(n=1,336)**  **Median (IQR)** | | | | | |
| --- | --- | --- | --- | --- | --- | --- | --- | --- | --- | --- | --- | --- | --- | --- | --- | --- | --- | --- | --- | --- | --- | --- | --- | --- |
|  | **Rural**  **(n= 575)** | | **Migrants**  **(n= 539)** | | **Urban**  **(n=772)** | | **Rural**  **(n=582)** | | **Migrants**  **(n=436)** | | **Urban**  **(n=439)** | | **Rural**  **(n=859)** | | **Migrants**  **(n=646)** | | **Urban**  **(n=371)** | | **Rural**  **(n=382)** | | **Migrants**  **(n=382)** | | **Urban**  **(n=572)** | |
|  | **V**  n=316 | **NV**  n=259 | **V**  n=323 | **NV**  n=216 | **V**  n=383 | **NV**  n=389 | **V**  n=207 | **NV**  n=375 | **V**  n=154 | **NV**  n=282 | **V**  n=151 | **NV**  n=288 | **V**  n=86 | **NV**  n=773 | **V**  n=78 | **NV**  n=568 | **V**  n=54 | **NV**  n=317 | **V**  n=105 | **NV**  n=277 | **V**  n=107 | **NV**  n=275 | **V**  n=184 | **NV**  n=388 |
| Energy kcal/day | 2147.6 (1703.2 to 2578.2) | 2083.0 (1658.8 to 2598.0) | 2837.6 (2413.4 to 3278.0) | 2814.8 (2426.5 to 3434.6) | 2851.8 (2474.4 to 3391.5) | 3031.8 (2568.2 to 3508.7)** | 3005.0 (2431.1 to 3520.0) | 3164.9 (2595.1 to3749.6)** | 3182.2 (2580.1 to 3735.5) | 3329.9 (2705.4 to3939.4) | 3209.1 (2651.4 to 3713.7) | 3375.4 (2780.6 to 3996.2)* | 2165.3 (1808.2 to 2575.4) | 2236.0 (1743.5 to 2845.8) | 2380.8 (1998.1 to 2901.4) | 2436.5 (2010.0 to 2997.6) | 2478.8 (1928.0 to 3109.8) | 2577.2 (2057.8 to 3192.9) | 2648.3 (2169.7 to 3257.7) | 3015.4 (2425.4 to 3764.2)** | 2605.1 (2350.6 to 3480.4) | 2637.6 (2198.3 to 3365.8) | 2658.3 (2207.1 to 3322.9) | 2786.4 (2326.3 to 3502.4)* |
| Protein g/day | 65.9 (53.3 to 77.7) | 68.4 (54.4 to 89.2) | 84.8 (73.9 to 99.6) | 90.3 (77.0 to 109.2)** | 85.7 (76.0 to 101.8) | 98.0 (82.8 to 114.5)** | 83.3 (68.1 to 96.4) | 92.1 (75.3 to 107.9)** | 84.5 (69.2 to 98.4) | 90.2 (73.1 to 106.6)** | 83.5 (70.1 to 98.1) | 90.9 (75.3 to 104.9)** | 51.8 (44.1 to 60.7) | 56.6 (43.8 to 72.9)* | 59.1 (49.7 to 71.7) | 69.4 (55.6 to 84.5) ** | 58.6 (47.6 to 74.2) | 71.9 (56.5 to 90.9) ** | 65.5 (54.1 to 81.3) | 83.5 (65.4 to 105.5)** | 68.0 (58.7 to 90.2) | 72.9 (60.2 to 92.4) | 67.9 (55.5 to 83.7) | 78.0 (64.5 to 99.7) ** |
| Carbohydrate  g/day | 323.2 (258.8 to 386.6) | 305.4 (247.8 to 382.1) | 453.3 (383.3 to 523.2) | 456.2 (388.6 to 540.5) | 457.1 (392.3 to 527.8) | 469.9 (396.5 to 540.6) | 489.9 (389.5 to 578.3) | 509.4 (414.5 to 597.5) | 505.4 (393.9 to 586.3) | 510.1 (411.4 to 599.1) | 511.3 (424.4 to 589.5) | 543.9 (432.0 to 624.5)* | 370.9 (302.5 to 432.6) | 362.5 (290.8 to 459.4) | 392.4 (328.2 to 462.) | 373.7 (308.7 to 458.4) | 393.5 (291.8 to 494.4) | 387.9 (316.0 to 485.7) | 439.5 (357.3 to 532.1) | 508.0 (383.3 to 608.3)** | 444.1 (367.4 to 552.3) | 421.1 (351.0 to 524.6) | 418.8 (351.4 to 503.3) | 436.8 (356.5 to 537.8 ) |
| Fibre g/day | 10.2 (8.0 to 12.8) | 9.6 (7.6 to 12.7) | 15.0 (12.8 to 18.2) | 15.0 (12.7 to 18.7) | 15.4 (12.8 to 18.8) | 15.6 (13.2 to 18.7) | 13.7 (11.1 to 17.2) | 14.5 (11.2 to 17.9) | 16.5 (13.1 to 19.9) | 16.5 (12.9 to 20.2) | 15.9 (12.8 to 18.3) | 17.1 (13.1 to 19.9) ** | 8.6 (7.1 to 11.5) | 8.4 (8.4 to 11.6) | 11.2 (9.0 to 15.8) | 10.6 (8.3 to 14.4) | 11.6 (8.1 to 15.4) | 11.6 (8.5 to 15.0) | 12.5 (9.0 to 18.0) | 16.1 (11.7 to 21.8) ** | 13.2 (10.7 to 17.0) | 13.4 (10.1 to 17.6) | 12.7 (10.1 to 16.2) | 13.6 (10.6 to 17.5) |
| Total fat g/day | 65.6 (49.7 to 82.0) | 63.7 (48.2 to 85.3) | 73.0 (61.1 to 88.2) | 73.8 (61.0 to 94.1) | 77.2 (62.3 to 94.5) | 83.4 (67.6 to 103.5)** | 73.1 (58.7 to 95.0) | 83.5 (65.7 to 105.3)** | 94.4 (72.6 to 120.0) | 103.0 (78.9 to 127.3) | 94.8 (70.8 to 111.1) | 99.4 (71.0 to 124.2)* | 57.0 (37.7 to 76.3) | 57.0 (41.9 to 75.9) | 69.0 (55.4 to 90.8) | 72.9 (57.0 to 94.1) | 74.2 (61.6 to 98.0) | 80.0 (59.3 to 100.4) | 69.7 (54.7 to 91.0) | 73.8 (54.7 to 98.6) | 75.5 (59.7 to 101.2) | 76.5 (56.6 to 102.9) | 74.9 (61.7 to 101.3) | 82.5 (64.1 to 110.5)* |
| Sat fat g/day | 21.8 (15.5 to 28.8) | 19.9 (14.5 to 27.4)* | 23.1 (17.9 to 28.5) | 21.9 (17.4 to 27.9) | 24.8 (19.3 to 31.3) | 25.7 (20.7 to 33.1) | 18.3 (13.3 to 25.1) | 20.0 (15.1 to 27.2)* | 22.3 (16.8 to 29.9) | 24.0 (18.0 to 31.3) | 23.6 (16.0 to 30.0) | 23.7 (16.3 to 35.4) | 16.2 (11.1 to 25.5) | 16.1 (11.5 to 23.1) | 18.9 (14.4 to 25.6) | 20.2 (15.2 to 26.7) | 22.6 (15.5 to 28.1) | 21.7 (15.9 to 29.1) | 25.8 (20.7 to 32.9) | 26.7 (20.6 to 34.4) | 25.4 (18.9 to 33.8) | 24.8 (18.7 to 35.0) | 25.5 (19.7 to 33.7) | 28.4 (21.0 to 38.8) ** |
| MUFA g/day | 27.2 (19.6 to 34.1) | 26.7 (19.9 to 35.3) | 28.5 (22.8 to 34.7) | 29.6 (23.7 to 38.4) | 27.4 (19.9 to 35.2) | 29.8 (21.5 to 39.6) ** | 19.8 (14.5 to 29.6) | 24.1 (18.3 to 33.1) ** | 27.1 (20.6 to 36.9) | 29.6 (22.8 to 42.4) | 22.8 (18.0 to 28.0) | 24.8 (18.5 to 31.1) | 15.1 (10.7 to 23.2) | 16.8 (11.7 to 23.1) | 16.7 (12.8 to 22.5) | 18.7 (14.4 to 25.0) | 18.5 (14.5 to 24.0) | 20.1 (14.7 to 27.2) | 19.2 (15.3 to 24.5) | 20.7 (15.2 to 28.6) | 19.5 (14.4 to 27.2) | 18.8 (13.2 to 26.3) | 18.6 (14.6 to 26.1) | 19.6 (15.4 to 28.0) |
| PUFA g/day | 10.5 (8.0 to 13.4) | 10.5 (8.1 to 14.4) | 13.6 (11.3 to 17.5) | 14.1 ( 11.5 to 18.0) | 15.4 (11.8 to 22.4) | 17.5 (13.0 to 25.7) ** | 26.2 (18.7 to 31.7) | 28.7 (19.4 to 38.0) ** | 33.7 (23.1 to 45.2) | 32.9 (23.8 to 46.3) | 36.1 (27.0 to 43.6) | 38.2 (28.1 to 46.2) | 16.9 (10.9 to 24.6) | 17.1 (11.0 to 26.0) | 26.8 (19.4 to 35.4) | 27.4 (19.9 to 36.8) | 29.4 (22.8 to 37.9) | 28.2 (20.4 to 38.1) | 18.3 (11.2 to 28.2) | 18.9 (13.1 to 27.0) | 26.5 (18.0 to 36.7) | 24.3 (18.0 to 34.6) | 26.4 (19.8 to 34.4) | 26.9 (21.1 to 35.6) |
| Iron mg/day | 19.4 (14.9 to 23.4) | 18.8 (15.0 to 23.3) | 30.0 (25.2 to 36.0) | 30.9 (25.9 to 37.6) | 30.2 (25.6 to 35.5) | 31.6 (26.6 to 37.0)* | 27.5 (22.7 to 32.5) | 28.7 (22.9 to 34.9) | 32.3 (26.4 to 37.9) | 33.0 (25.7 to 38.7) | 30.7 (25.8 to 34.9) | 32.8 (26.4 to 37.5)* | 12.6 (9.8 to 16.0) | 12.7 (9.5 to 17.0) | 18.5 (14.9 to 23.3) | 18.4 (14.2 to 23.3) | 20.3 (15.1 to 25.3) | 19.5 (14.5 to 25.4) | 22.2 (16.9 to 28.9) | 25.2 (18.6 to 33.7)* | 20.9 (17.2 to 26.1) | 20.3 (15.8 to 26.3) | 19.9 (16.1 to 24.4) | 20.7 (16.7 to 27.6) |
| Calcium mg/day | 906.4 (667.4 to 1123.3) | 851.8 (637.9 to 1170.6) | 1011.2 (828.6 to 1243.1) | 1052.3 (811.4 to 1287.3) | 1134.6 (896.7 to 1413.4) | 1156.2 (955.5 to 1514.2)* | 764.4 (580.9 to 1000.7) | 857.5 (647.4 to 1060.8)** | 898.3 (665.9 to 1090.3) | 948.8 (748.5 to 1173.8)* | 887.8 (644.7 to 1046.6) | 923.7 (675.4 to 1114.2) | 595.4 (468.8 to 798.4) | 638.4 (438.6 to 873.3) | 903.6 (639.7 to 1179.8) | 881.2 (679.5 to 1183.1) | 990.1 (743.8 to 1268.2) | 904.8 (688.1 to 1214.1) | 1117.4 (824.1 to 1498.2) | 1395.0 (1056.8 to 1800.5)** | 1156.5 (966.6 to 1520.2) | 1176.4 (896.8 to 1585.4) | 1157.5 (899.4 to 1441.4) | 1221.6 (949.5 to 1555.2) |
| Zinc mg/day | 6.4 (5.0 to 8.3) | 6.4 (4.9 to 8.8) | 13.4 (11.3 to 15.5) | 13.9 (11.8 to 16.7) | 13.5 (11.7 to 16.0) | 14.6 (12.3 to 16.8) ** | 12.5 (10.3 to 14.8) | 13.7 (11.1 to 16.1) ** | 13.6 (11.2 to 16.0) | 14.5 (11.3 to 17.0)* | 13.5 (11.2 to 15.8) | 14.7 (12.3 to 17.0) ** | 8.3 (6.9 to 10.1) | 8.8 (7.0 to 11.5) | 9. 1 (7.8 to 11.0) | 10.0 (8.2 to 12.4)* | 9.1 (7.2 to 11.9) | 10.3 (8.4 to 12.7)* | 10.9 (9.0 to 13.8) | 13.8 (10.9 to 17.4) ** | 10.7 (9.3 to 14.1) | 11.2 (9.2 to 14.1) | 10.3 (8.7 to 12.8) | 11.6 (9.7 to 14.7) ** |
| Vit C mg/day | 100.9 (69.5 to 135.9) | 93.4 (69.2 to 138.0) | 144.8 (111.5 to 188.9) | 138.0 (105.7 to 191.5) | 154.5 (114.4 to 212.9) | 151.1 (113.1 to 203.7) | 123. 2 (84.6 to 173.3) | 129.0 (91.8 to 168.8) | 183.6 (125.1 to 250.6) | 187.4 (126.2 to 233.5) | 170.0 (125.4 to 224.8) | 189.4 (122.6 to 237.8) | 95.8 (69.5 to 158.9) | 93.6 (62.8 to 147.3) | 170.4 (126.6 to 259.0) | 144.3 (100.7 to 222.8)** | 172.5 (119.8 to 287.2) | 160.0 (104.1 to 216.6) | 100.7 (69.0 to 132.9) | 101.8 (74.2 to 151.8) | 150.6 (122.1 to 213.8) | 154.4 (115.9 to 205.3) | 158.4 (133.2 to 221.4) | 170.2 (125.4 to 225.9) |
| Vit B12 mcg/day | 1.4 (0.9 to 1.9) | 2.1 (1.4 to 3.3) ** | 1.3 (0.9 to 1.8) | 1.9 (1.3 to 2.7) ** | 1.6 (1.2 to 2.2) | 2.4 (1.7 to 3.5) ** | 0.7 (0.5 to 1.1) | 1.6 (0.9 to 2.6) ** | 0.7 (0.5 to 1.1) | 1.5 (0.9 to 2.2) ** | 0.8 (0.5 to 1.2) | 1. 2 (0.9 to 1.9) ** | 0.9 (0.6 to 1.4) | 2.2 (1.3 to 4.2) ** | 1.7 (1.2 to 2.9) | 3.8 (2.4 to 6.8) ** | 2.0 (1.3 to 3.3) | 3.8 (2.2 to 6.4) ** | 1.3 (0.9 to 1.8) | 1.9 (1.2 to 2.9) ** | 1.3 (1.0 to 1.9) | 1.9 (1.3 to 2.7) ** | 1.4 (1.0 to 1.9) | 2.2 (1.6 to 3.3) ** |
| Folate mcg/day | 307.4 (232.5 to 374.7) | 288.3 (228.7 to 382.6) | 387.2 (324.3 to 465.1) | 389.4 (322.4 to 471.2) | 406.2 (343.6 to 481.5) | 417.5 (354.2 to 501.4) | 335.2 (267.8 to 401.3) | 343.0 (276.7 to 414.1) | 380.1 (300.9 to 452.5) | 386.6 (308.1 to 457.8) | 366.4 (299.2 to 433.4) | 388.4 (308.0 to 446.8) | 242.4 (180.7 to 315.4) | 243.3 (182.4 to 316.6) | 289.4 (221.0 to 382.5) | 272.1 (209.1 to 354.4) | 308.4 (238.2 to 385.9) | 296.3 (219.1 to 363.4) | 284.1 (213.9 to 368.8) | 300.6 (221.4 to 393.5) | 367.7 (294.0 to 476.1) | 356.5 (285.2 to 466.8) | 364.8 (293.6 to 461.1) | 387.6 (306.8 to 476.6) |

**p<0.01 *p<0.05 (Wilcoxon Rank Sum non-parametric) **V** - Vegetarians **NV** - Non-vegetarians
